# *MED12*-Related (Neuro)Developmental Disorders: A Question of Causality

**DOI:** 10.3390/genes12050663

**Published:** 2021-04-28

**Authors:** Stijn R. van de Plassche, Arjan P. M. de Brouwer

**Affiliations:** Department of Human Genetics, Donders Institute for Brain, Cognition and Behavior, Radboud University Medical Center, 6500 HB Nijmegen, The Netherlands; stijn.vanderplassche@radboudumc.nl

**Keywords:** MED12 variants, Hardikar syndome, intellectual disability, iNeuronal model, transcription regulation, expression profile

## Abstract

MED12 is a member of the Mediator complex that is involved in the regulation of transcription. Missense variants in MED12 cause FG syndrome, Lujan-Fryns syndrome, and Ohdo syndrome, as well as non-syndromic intellectual disability (ID) in hemizygous males. Recently, female patients with de novo missense variants and de novo protein truncating variants in MED12 were described, resulting in a clinical spectrum centered around ID and Hardikar syndrome without ID. The missense variants are found throughout MED12, whether they are inherited in hemizygous males or de novo in females. They can result in syndromic or nonsyndromic ID. The de novo nonsense variants resulting in Hardikar syndrome that is characterized by facial clefting, pigmentary retinopathy, biliary anomalies, and intestinal malrotation, are found more N-terminally, whereas the more C-terminally positioned variants are de novo protein truncating variants that cause a severe, syndromic phenotype consisting of ID, facial dysmorphism, short stature, skeletal abnormalities, feeding difficulties, and variable other abnormalities. This broad range of distinct phenotypes calls for a method to distinguish between pathogenic and non-pathogenic variants in MED12. We propose an isogenic iNeuron model to establish the unique gene expression patterns that are associated with the specific MED12 variants. The discovery of these patterns would help in future diagnostics and determine the causality of the MED12 variants.

## 1. Introduction

MED12 is a member of the multi-protein Mediator complex. The Mediator complex is part of the preinitiation complex, and is involved in transcriptional regulation [1]. It functions as a switchboard, relaying signals from gene-specific regulatory proteins to RNA polymerase II, to regulate transcription initiation and transcription elongation [2]. Mediator is also involved in other cellular processes such as chromatin folding and looping, as well as regulation of gene expression by interaction with non-coding RNAs (ncRNA) [3,4].

**Figure 1:**
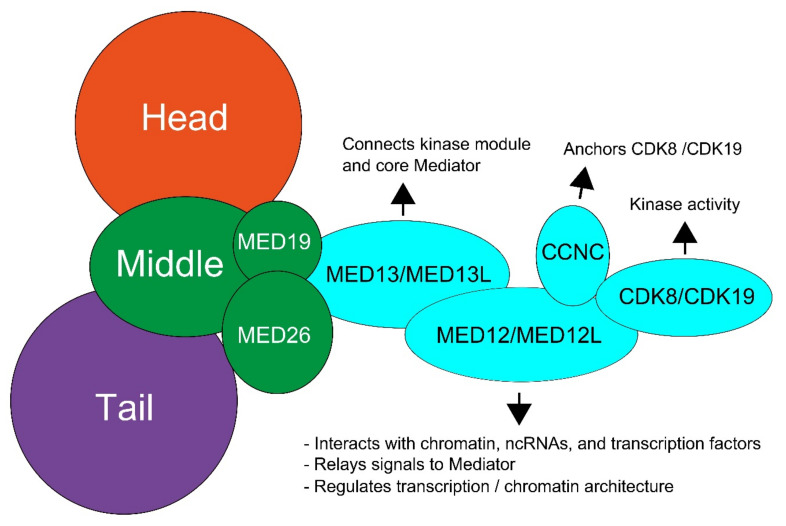
Schematic overview of the structure of the Mediator kinase subunit and the functions of individual proteins. The kinase subunit (light blue) consists of MED13, MED12, Cyclin C (CCNC), and cyclin-dependent kinase 8 (CDK8). Paralogs have been discovered for all subunit components expect CCNC. These paralogs are MED13L, MED12L, and CDK19. Adjusted from Aranda-Orgilles et al. [2].

The “core” Mediator complex contains 31 subunits, consisting of the head, middle, and tail modules, while the kinase module can reversibly associate with this “core” complex [2] (Figure 1). The kinase module consists of MED12, MED13, Cyclin C (CCNC), and cyclin-dependent kinase 8 (CDK8), and the paralogs MED12L, MED13L, and CDK19. The paralogs are mutually exclusive with each other, but not with other kinase subunits, allowing for eight different kinase modules [5,6,7]. The different kinase modules are cell type-specific, as MED12 and MED13 are ubiquitously expressed [8], whereas MED13L is mostly expressed in brain and heart tissue [9], and MED12L is mostly expressed in the brain [10]. CDK8 and CDK19 seem to have similar expression patterns and are also ubiquitously expressed [11,12].

MED12 interacts with DNA-bound transcription factors and hence acts as a hub for the communication of transcription factors with the kinase subunit and core Mediator [8]. It also regulates the activity of the kinase module via interaction with a conserved T-loop on CDK8 [13]. Due to the important role of MED12 in the regulation of several developmental and signaling pathways, somatic variants in *MED12* are known to be involved in cancer and germline variants cause a spectrum of developmental disorders that involve Hardikar syndrome to relatively unspecific non-syndromic intellectual disability (ID) [8,14,15,16].

## 2. Variants in *MED12* Cause (Neuro)Developmental Disorders

**Figure 2:**
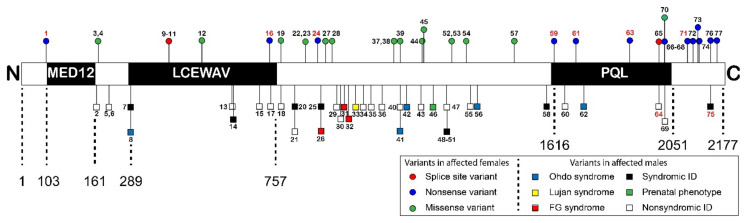
Schematic overview of variants distributed across the predicted functional domains in the MED12 protein (not to scale). Previously described ID associated hemizygous variants found in males are presented below the protein scheme. Patient numbers in red describe nonsense mutations. Above the protein scheme all known variants in females are described. Patient numbers in red describe patients with Hardikar syndrome. The MED12 domain that is part of the kinase section of Mediator, the LCEWAV domain with no known function, and the catenin-binding PQL domain are indicated. Only domains predicted by the PFAM database (https://pfam.xfam.org/ (accessed on 1 April 2021) were included in this figure. Figure was adapted from [16] and supplemented with data from [17]. Descriptions of individual patients can be found in Table S1.

The disorders caused by variants in *MED12* are referred to as *MED12*-related disorders. *MED12* is located on Xq13.1. Most patients are male who have inherited the missense variant from a unaffected carrier female (Table S1) [17]. Several X-linked developmental syndromes are associated with hemizygous *MED12* variants, including Opitz–Kaveggia syndrome (FG syndrome, MIM 305450), Lujan–Fryns syndrome (MIM 309520), and X-linked Ohdo syndrome (MIM 300895), as well as non-syndromic X-linked ID [18]. The three syndromes are symptomatically similar, as they encompass intellectual disability, hypotonia, macrocephaly, hyperactivity, abnormalities of the corpus callosum, behavioral abnormalities, and typical facial features (Table 1) [19]. These three syndromes are caused by only eight out of the 77 of the *MED12* variants that are known to cause disease. Other variants have been described to cause syndromic, but not fitting exactly any of the three syndromes, or non-syndromic ID. The missense variants responsible for these disorders are located throughout *MED12* and cannot be found in one specific protein domain or motif (Figure 2).

Recently, females with *MED12* variants were identified as well [16,17]. Remarkably, besides missense variants, nonsense and frameshift variants were found. Protein truncating variants have been found in only two men, and then only in the last amino acid residues at the end or after the PQL domain at the C-terminus [20,21]. Li et al. [16] describe a cohort of seven female patients with Hardikar syndrome, a rare multiple congenital anomaly syndrome characterized by facial clefting, pigmentary retinopathy, biliary anomalies, and intestinal malrotation. Hardikar syndrome does not include ID and does not present with other symptoms often associated with MED12-related disorders. All the females harbor de novo MED12 nonsense or frameshift variants mostly in the N-terminus of MED12, which are predicted to be subject to nonsense-mediated decay [16]. Furthermore, Polla et al. [17] describe a cohort of 18 females with syndromic and non-syndromic ID, all with de novo MED12 variants, five of which were nonsense variants, two were splice-site variants, and 11 were missense variants [17]. The nonsense variants were located at either the very end of the PQL domain or after the PQL domain in the C-terminal part of MED12. The protein truncating variants were associated with a severe, syndromic phenotype consisting of ID, facial dysmorphism, short stature, skeletal abnormalities, feeding difficulties, and variable other abnormalities. Of the 11 de novo missense variants, ten were located in between the LCEWAY and PQL domains. These variants were associated with a less specific but homogeneous phenotype including severe ID, autistic features, limited speech and variable other anomalies, overlapping both with females with truncating variants as well as with males with missense variants.

There is a distinct discrepancy in the phenotype caused by *MED12* protein truncating variants in females. On the one hand, these cause Hardikar syndrome without ID; on the other hand, they cause syndromic ID. There is evidence that this could be due to a stochastic event in females, where X-inactivation (XCI) of the allele carrying the mutant variant in brain tissue determines whether truncating variants result in Hardikar or an ID disorder. This is supported by the fact that XCI in fibroblasts was not correlated with symptom severity in several studies [17,19,20]. We therefore propose that XCI in blood cannot be used as proxy for XCI in the brain. Additionally, siRNA-mediated knockdown experiments, which imitate a truncating variant being subject to nonsense-mediated decay, lead to disruption of developmental pathways similar to that observed for missense variants [22,23,24,25,26]. This suggests that it is not the position of the variant that determines phenotype, but the effect of the variant on MED12 activity. This would indicate that skewing of XCI in specifically the brain dictates the neurological phenotype.

The missense variants that cause disease in males and females are not clustered in one specific protein domain or motif and can cause different severity of (neuro)developmental disease in males and females (Figure 2). In addition, the truncating variants have been described in females and two males with divergent phenotypes, and they might be subject to NMD. Most of these are de novo, but two are not, which complicates matters. There is a clear need for a relatively high throughput model discounting X-inactivation to investigate the variants to determine whether a variant is causative.

## 3. MED12 Functional Domains

**Figure 3:**
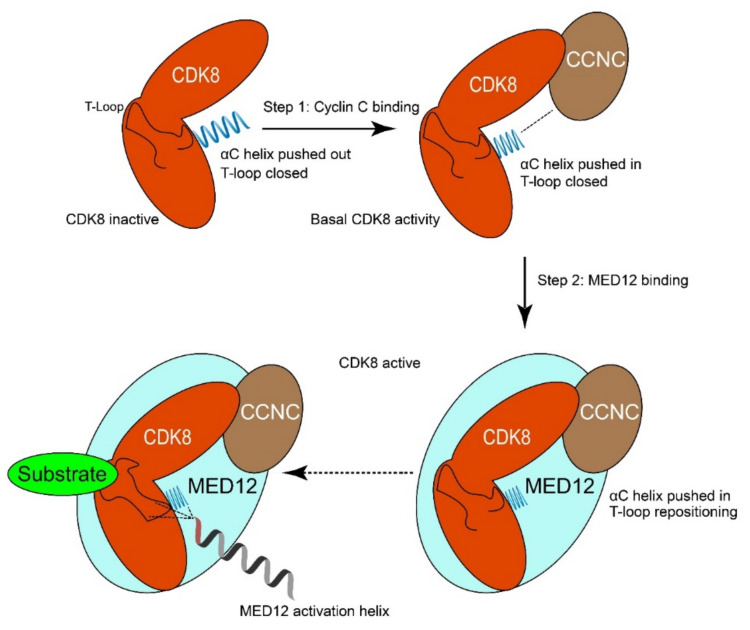
Model of how MED12 activates CDK8. Step 1: Cyclin C binds to CDK8 and pushes the αC-helix of CDK8 into the “pushed-in” conformation. This binding event is crucial for the formation of the active site of CDK8 and results in basal kinase activity. Step 2: MED12 binding to CDK8/CCNC stabilizes and activates the MED12-CCNC-CDK8 complex. In particular, an activation helix in MED12 contacts and stabilizes the T-loop of CDK8, thereby activating the kinase. Moreover, MED12 binding favors remodeling of the active site of CDK8 to enhance its activity. Adapted from Klatt et al. [13].

The MED12 protein has several functional domains. The most N-terminal domain is the MED12 domain (transcription mediator complex subunit Med12) (amino acid residue 103–161), which interacts with CDK8-CCNC [13]. This domain is responsible for the activation of the CCNC-CDK8 kinase activity by wrapping around the entire CDK8 protein (Figure 3) [27]. This would place MED12 residues 30 to 42 in close proximity with the conserved T-loop required for CDK8 activation. Variant hotspot residues often found to be mutated in uterine leiomyoma, such as Leu36, Gln43, and Gly44, are part of this activation helix. The activation helix is essential for the ability of MED12 to activate CDK8 kinase activity, although it is not essential for binding to the CCNC-CDK8 complex. This model, in which precise positioning of the conserved MED12 activation helix is required for activation of CDK8 via its T-loop, could explain the frequent occurrence of variants in cancers, as this would directly influence the ability of MED12 to activate CDK8 [8,13].

The LCEWAV domain (amino acid residues 289–757) is located between the MED12 and PQL domains. The domain name is derived from a conserved sequence motif LCEWAV. To date, no functions of the LCEWAV domain have been described, although the presence of a number of pathologic variants in this domain (Figure 2) suggests that it might have some undiscovered function [8].

The most C-terminal domain is the PQL domain (amino acid residues 1616–2051). This domain is named after its composition, which consists mainly of proline, glutamine, and leucine amino acid residues [22]. The MED12 PQL domain is the interface for four transcriptional pathways, SOX9 signaling, β-catenin signaling, the REST (RE1-silencing transcription factor) pathway and the sonic hedgehog (SHH) pathway.

The PQL domain is essential for signaling of the SOX9 pathway, a developmental pathway that is involved in chondrocyte differentiation and male sex determination [28]. In a yeast two-hybrid assay, interaction between SOX9 and MED12 depended on the presence of an intact PQL domain. These results were later confirmed in zebrafish, as TRAP230 (a zebrafish MED12 ortholog) mutants lacking the PQL domain exhibited a decrease in SOX9 signaling compared to wild-type animals [29]. In humans, it seems unlikely that the interaction with SOX9 plays an important role in the effects of the variants resulting in intellectual disability as chondrocyte differentiation and male sex determination are seemingly normal. However, since SOX9 is also thought to play a role in cholangiocyte development [30], it may be involved in the formation of the biliary tree, and therefore be relevant to Hardikar Syndrome.

PQL is also the domain where β-catenin interacts with Mediator [22]. The Wnt/β-catenin pathway is a developmental pathway important in the regulation of cell fate and embryonic development. Ectopic expression of the PQL domain was reported to inhibit transactivation of β-catenin in a dose-dependent manner. Conversely, siRNA-mediated knockdown of MED12 inhibited the transcriptional activity of β-catenin, while expression of PQL alone inhibited β-catenin in a dominant-negative fashion [22]. This indicates that the PQL domain is required for MED12 to transduce β-catenin signaling. These results were later independently confirmed by two different studies that showed a similar effect on β-catenin after MED12 knockdown [23,31]. Zhou et al., observed a five-fold decrease in β-catenin transactivation activity after siRNA mediated knockdown of MED12. Furthermore, a MED12 mutant lacking the PQL domain was unable to rescue β-catenin transactivation activity in cells depleted of endogenous MED12 [23].

The PQL domain is also involved in the REST (RE1-silencing transcription factor) pathway [23]. A network of functional interactions between MED12, G9a, and REST was established via immunoprecipitation [23,32]. REST is a master transcriptional regulator of neuron-specific genes in neurogenesis and neuronal differentiation [33]. It also plays a role in fine-tuning of genes involved in synaptic plasticity during postnatal brain development. Deletion of the PQL domain lowered the silencing activity of REST [23]. Missense variants in this domain could influence normal regulation of gene expression in neuronal cells.

Lastly, the PQL domain binds to Gli3 that is a key controller of the SHH pathway [31]. By binding to Gli3, MED12 negatively regulates the SHH pathway. This is supported by the fact that SHH signaling increased upon knockdown of MED12. Increased SHH signaling after MED12 knockdown is consistent with later reports that identify MED12 as an inhibitor of the SHH pathway [8,24]. As SHH is essential for the specification of oligodendrocytes, proliferation of neural precursors and control of axon growth [34], missense variants in PQL could influence any of these cellular processes.

Two other regions of MED12 are not predicted by PFAM, but often recognized as well. These regions are the LS region (amino acid residues 501–1650) and the C-terminal opposite paired (OPA) region (amino acid residues 2087–2212) [28]. The LS region takes up a large part of the MED12 gene and is also where most of the pathogenic variants are found (Figure 2). To our knowledge, no specific function has been attributed to the LS region to date. However, both the p.Arg961Trp variant found in FG syndrome and the p.Asn1007Ser variant found in Lujan syndrome are involved in the silencing activity of REST, indicating that these variants in the LS domain affect the relay of signals through MED12. The p.Arg961Trp and the p.Asn1007Ser variant were also shown to disrupt Gli3-dependent SHH signaling [8,24]. The variants were shown to impair activation of CDK8, while not affecting Gli3 and G9a binding [24,25]. This might mean that the LS region is important for regulating structural changes in response to transcriptional signals.

The most C-terminal region, OPA, has also not been connected to specific functions of MED12. One research group showed that MED13 requires amino acid residues 1616–2177, encompassing both the PQL domain and the OPA region, to bind to MED12 [35]. Unfortunately, no follow-up experiments that made a distinction between a possible function of either the PQL or OPA were reported. It seems reasonable to speculate that the transcription factor-binding PQL domain might not function as a binding platform for MED13 at the same time, leaving OPA as the MED13 binding partner. Alternatively, binding of MED12 to interfacing transcription factors could be followed up by MED13 binding to relay the signal to core Mediator, leaving OPA without a function. Follow-up research would be needed to confirm the specific role that OPA plays in MED13 binding. Interestingly, in-frame insertions or deletions (+12 bp or −15 bp) within the OPA region are known as a risk factor for psychosis, a symptom that is not associated with other MED12 variants [36,37]. Overall, very little structural and functional data are available for the LS and OPA regions, making this a possible field on which to focus future study.

## 4. MED12 Regulates Gene Expression by Binding to (Super-)Enhancers and ncRNA

Mediator functions as a bridge between cell type-specific transcription factors and the transcriptional machinery. As such, Mediator complex, and specifically MED12, is increasingly found to have an important role in the regulation of (super-)enhancers [1]. Super-enhancers are clustered enhancers whose combined influence allows them to drive high levels of gene expression. They are characterized by their high occupancy of cell type-specific transcription factors, the presence of histone mark H3K27ac, and a high level of Mediator [38]. Loss of MED12 impacts the ability of super-enhancers to function, reducing their capacity to induce gene expression by approximately a half [38]. Mediator is required for the functional and physical interaction between enhancers and promoters [39]. Using a short hairpin RNA knockdown screen, *MED12* was identified, along with several other subunits of the Mediator complex as important for the maintenance of *OCT4* expression in mouse embryonic stem cells. Factors important for DNA looping, such as Cohesin and Nipbl, were also identified in the same screen. Subsequent chromatin conformation capture verified that looping between enhancer and promoter regions was dependent on the presence of MED12. Recruitment of MED12 to enhancer sites is likely cell type-specific, as mouse embryonic fibroblasts showed a different pattern of gene activation compared to the embryonic stem cells [39]. Cell type-specificity of MED12 is supported by independent findings in other cell types [2,40,41].

The role of MED12 and the kinase module in the regulation of the cell state was further investigated by using human hematopoietic stem cells (HPSCs) [2]. First, conditional *MED12* knockouts were induced in mice embryos, allowing for a knock-down of *MED12* at embryonic day 10.5, which resulted in mortality within two weeks after birth, as well as aberrant haematopoiesis. The effect was cell type-specific, as a similar knockout in mouse embryonic fibroblasts did not affect cell viability. The affected function of MED12 was likely independent of its involvement with the kinase subunit, as knockdown of *MED13*, *CDK8*, and most importantly *CCNC* did not recapitulate the phenotype. Unfortunately, *MED13L* knockdown was not performed, as this would have allowed distinction between a truly MED12-specific function or an effect through the interaction with core Mediator. The expression of HPSC-specific genes was disrupted after loss of MED12, indicating that MED12 is important in maintaining a cell type-specific expression pattern. Through an unbiased analysis of enhancer regions, it was discovered that MED12 co-localizes to a high degree with P300 on active enhancer regions, where P300 was not found after *MED12* knockdown. P300 is involved in regulation of H3K27ac, a histone marker of active enhancers, and this may be the mechanism through which MED12 preserves enhancer activity, without having to invoke the kinase function of the subunit. In this way, MED12 is hypothesized as an essential platform for communication between co-activators, transcription factors, and the Mediator complex required to maintain the active state of enhancers. In support of this hypothesis, similar results were obtained when investigating transcriptional dysregulation in uterine leiomyoma samples with *MED12* variants [42]. In this study, uterine leiomyoma tissue samples from 15 women with confirmed MED12 p.Gly44Asp or p.Gly44Ser variants were analyzed for epigenetic changes. Differential gene expression analysis identified 5831 differentially regulated genes, as well as a significant number of regions with changes in H3K27ac occupancy, thus evidencing differential enhancer usage in patients with a *MED12* variant.

MED12 also mediates the interaction between non-coding RNAs (ncRNAs) and Mediator. There is some evidence that Mediator, and specifically MED12, is required for the activation of neighboring genes by a class of ncRNAs, called ncRNAs activating (ncRNA-a) [43]. Deletion of *MED12* resulted in loss of activation of transcription after induction of several ncRNA-as. MED12 also was vital for the formation of chromatin loops between the ncRNA-a and its target genes, similar to the role of MED12 on enhancers [39]. MED12 is also involved with another kind of ncRNAs, which are derived from enhancers and are thus called enhancer RNAs (eRNA) [44]. Interestingly, in breast cancer cells eRNA production was found to be dependent on both JMJD6 and MED12 [40]. As eRNA has been reported to be important for transcriptional activation, this may be another way in which MED12 plays a role in enhancer control [45].

## 5. MED12 Variants Show That the Protein Can Function as a Switchboard of Sorts

How can *MED12* variants result in the diverse clinical phenotypes that are observed in these disorders? One hypothesis is that MED12 functions as a switchboard of sorts, where it relays information from specific transcription factors to the appropriate outcome. A variant in *MED12* could lead to the loss of signal transduction for some signals, but not others, as not all signals will be integrated on the same part of the protein. In support of this notion, research into specific variants has consistently shown that variants can influence one pathway that is related to MED12, while not affecting others.

Research into the effect of MED12 on specific regulatory pathways found that MED12 is required for the functioning of the REST pathway via its interaction with G9a and REST, which both are involved in the suppression of neural genes in non-neural cells [23,32]. Disruption of the REST pathway lead to the unscheduled de-repression of REST target genes, which could negatively affect neuronal differentiation [23,32]. G9a interacts directly with the MED12 PQL domain. G9a-MED12 then forms a three-way interaction with REST. Interestingly, knockdown of *CDK8* and subsequent loss of CCNC did not result in a similar phenotype, confirming a kinase-independent function of MED12. Importantly, the p.Arg961trp and p.Asn1007Ser variants failed to rescue REST silencing after knockdown of wild-type *MED12*, but these mutant proteins were still able to rescue β-catenin activity. It is probable that this distinction derives from a function of the mutated LS region, since both G9a and β-catenin interact with the unaffected PQL domain. The recruitment of MED12, G9a, and other Mediator components to RE1 elements was significantly impaired in the MED12 mutants, pointing to specific disruption of the REST pathway. Similar results were obtained for p.Arg1148His and p.Ser1165Pro variants, which also showed impaired REST activation without influencing MED12 stability or G9a binding [26].

Gli3, a member of the SHH pathway, also interacts directly with the MED12 PQL domain [31]. In this context, MED12 was shown to functionally repress expression of SHH target genes, which was reversed upon binding of Gli3. MED12 p.Arg961Trp and p.Asn1007Ser were unable to rescue the inhibition of SHH signaling in MED12 null mice, but wildtype MED12 was [24]. Again, both variants were able to rescue β-catenin signaling. Interestingly, knockdown of *CDK8* resulted in a similar phenotype, suggesting that the role of MED12 in the regulation of the SHH pathway works via CDK8 kinase activity. Loss of CDK8 recruitment was observed on Gli3 binding sites in the variant but not the wildtype cells. It was found to be pathway-specific, as variant MED12 could still recruit CDK8 to PPARγ-target genes. This suggests that the variant protein loses the ability to respond correctly to specific signals, while not affecting other, distinct, signaling pathways. The evidence for involvement of the SHH pathway in MED12-related disorders was further broadened by the finding that variants p.Asn898Asp, p.Arg1214Cys, and p.Arg1295His also result in an increase of SHH signaling [25].

Recently, it was shown that specific *MED12* variants have a differential effect on immediate early gene (IEG) expression [41]. Several different variants, p.Arg206Gln, p.Asn898Asp, p.Arg961Trp, p.Asn1007Ser, p.Arg1148His, p.Ser1165Pro, and p.Arg1295His were investigated for their influence on *JUN*, *FOS*, and *EGR1* expression. Knockdown of *MED12* reduced the expression of IEGs, exemplifying that MED12 is involved in the regulation of IEG expression. Interestingly, the p.Arg1295His variant caused lower recruitment of MED12, CDK8, and RNA pol2 only on the *JUN* promoter, while not affecting the *FOS* and *EGR1* promoters. Similarly, the other five variants studied all resulted in a unique recruitment pattern, proving that these variants all influence MED12 recruitment to this set of genes in their unique way.

The variants that have been studied so far are all located in the LS region of MED12, which has no known function. They can influence both MED12 recruitment and recruitment of cofactors such as G9a and CDK8 [23,24,41]. This could mean that variants in *MED12* result in lower MED12 recruitment to its target genes, which in turn results in lower MED12-mediated recruitment of cofactors. The p.Arg961Trp and p.Asn1007Ser variants disrupt REST signaling in a kinase-independent manner, while disruption of the SHH pathway by the same two variants was dependent on CDK8 activity. It is thus likely that disruption of these pathways stems from a function specific to the LS region, where the variant does not necessarily influence the capacity of MED12 to activate CDK8. Such a function could be that the LS region is important for proper recruitment of MED12 in response to specific signals. In line with this hypothesis, the p.Arg961Trp and p.Gly958Glu variants in the LS region were unable to respond to specific nc-RNA signals, which are thought to be involved in recruitment of MED12 to its target genes [43].

**Figure 4:**
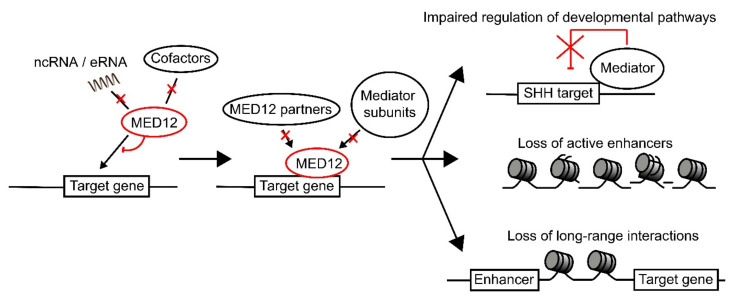
Model for the pathogenic effect of *MED12* variants. MED12 recruitment to its target gene is regulated via interaction with ncRNAs, eRNAs, and cofactors such as REST. We propose that specific mutations in MED12 disrupt its interactions with either ncRNAs, eRNAs, or cofactors, depending on where the mutation is present in the protein. This leads to reduced recruitment of MED12 to the target genes, which will in turn result in lower MED12-mediated recruitment of additional factors, such as G9a, as well as other Mediator subunits. This will lead to loss of regulation over developmental pathways, loss of regulation over chromatin state (leading to loss of active enhancers), as well as loss of long-range transcriptional interactions.

Overall, there is evidence that specific variants in MED12 exert their pathogenic effects through disruption of pathways specific to that variant. A unique pattern of disruption associated with specific *MED12* variants could help explain why there is such a large clinical variation in *MED12*- related disorders. Additionally, the presence of a large number of *MED12* variants in the LS region suggest that this region could have an as of yet undiscovered function, such as in the recruitment of MED12 to its target genes. We hypothesize that all *MED12* variants cause the mutant MED12 protein to lose the ability to respond to specific signaling factors [23,24,43], leading to impaired recruitment of MED12 and MED12-dependent recruitment of downstream factors [2,40] (Figure 4).

## 6. The Pathogenic Variants in Other Kinase Unit Proteins Cause a Similar Phenotype

*MED12* variants are responsible for several different ID syndromes, as well as non-syndromic ID. The phenotypes of these syndromes largely overlap, creating a spectrum of possible symptoms that can be caused by *MED12* variants. Interestingly, recent research has pointed out that variants in other proteins in the kinase subunit are also associated with syndromes involving ID, for which the symptoms fit into the spectrum of *MED12*-related disorders [46].

To date, 69 patients carrying de novo *MED13L* variants have been described (35 females, 34 males) [47]. All of these patients showed ID and developmental delay, and other aspects of the phenotypic spectrum of *MED12*-related disorders were also found, but to a lesser degree (Table 1). Variants were spread out over *MED13L*. Large deletions were detected in about 25% of patients. Loss-of-function variants were the most common sequence variant explaining roughly 75% of the patients. Missense variants tended to result in a more severe phenotype than other variants, suggesting these exert their effect in a dominant-negative way [9]. Missense variants clustered in exons 15–17 and 25–31, which are two highly conserved regions of both *MED13L* and *MED13* [9]. Despite high levels of conservation, it is not quite clear what the function of these domains is. Likely they involve the interaction with either core Mediator via MED19, or interaction with MED12(L), as these are thought to be the two main functions of MED13(L) [48].

De novo pathogenic variants were also found in *MED13*, and patients carrying these variants also exhibited symptoms that fit the spectrum seen for *MED12* [48] (Table 1). Other than observed in *MED13L*, *MED13* variants tended to cluster around a conserved phosphodegron motif in the N-terminus of the protein. This phosphodegron motif is recognized by SCF-Fbw7 ubiquitin ligase, which is important for the regulation of the level of MED13(L) [7]. This may be an important checkpoint for the regulation of the entire kinase module, as it was found that only MED13(L) bound to core Mediator was targeted for degradation [7].

Variants in *CDK8* were recently also found to cause a syndromic developmental disorder [49]. Calpena et al. [49] describe eight different de novo missense variants in 12 unrelated patients. Affected individuals presented with overlapping phenotypes characterized by hypotonia, mild to moderate ID, behavioral disorders, and variable facial dysmorphism, as well as agenesis of the corpus callosum (Table 1). All variants affected the CDK8 kinase domain, which is encoded by amino acids 21–335, and clustered specifically around the ATP binding pocket. Experiments investigating the kinase activity of the mutated CDK8 showed that phosphorylation of a well-characterized CDK8 target, STAT1, was similar to that of a catalytically inactive CDK8 mutant. These variants are hypothesized to work via a dominant-negative model, as the supply of active kinase modules is thought to be rate-limiting, and incorporation of a mutant CDK8 protein that is essentially catalytically dead would limit kinase module supply by 50% [49]. Similar to the findings for *CDK8*, de novo variants in the kinase domain of CDK19 were found in three unrelated patients presenting with an ID syndrome (Table 1) [5].

Seven patients with de novo variants in *MED12L* have been described to date [10]. All patients had a protein truncating variant and presented with symptoms fitting the spectrum expected for variants in *MED12* (Table 1). The three female patients carried variants that were predicted to result in a premature stop codon in the C-terminal part of the protein, as was also observed in several of the patients described by Polla et al. [17]. As the *MED12* C-terminal region is conserved between MED12L and MED12, this finding can be seen as independently confirming the causal effect of the variants described by Polla et al. [17].

Taken together, there is evidence that variants in most of the proteins involved in the kinase subunit result in a very similar phenotypic spectrum [46,50]. It might be worthwhile to consider that these proteins interact in similar processes, explaining why their symptoms overlap. Difference in symptoms per protein may be explained by divergent individual cellular functions [46,49,50].

## 7. Conclusions: A Question of Causality

MED12 is an important protein in the regulatory machinery of transcription and as such, variants to this gene can affect a large range of different processes. Male germline variants in *MED12* are involved in the X-linked intellectual disability syndromes FG syndrome, Lujan-Fryns syndrome, and Ohdo syndrome, as well as in non-syndromic intellectual disability. How variants in *MED12* lead to these specific, distinct syndromes is currently an open question. The recent discovery of two additional female cohorts that include several nonsense and frameshift variants that could result in truncating proteins or nonsense-mediated decay further obfuscates this problem [16,17].

There is evidence that specific variants can affect different pathways, such as developmental pathways, the stability of (super-)enhancers, as well as maintenance of transcription-permissive chromatin states [23,26,41,51]. However, no clear patterns differentiating the different syndromes have been discovered. Furthermore, most of the research regarding the effects of specific variants focusses on missense variants. It is likely that haploinsufficiency due to nonsense or frameshift variants can also result in disruption of developmental pathways, as deregulation of pathways was observed in several independent experiments where *MED12* was knocked down [23,24]. Additionally, it is unclear whether specific *MED12* variants can influence enhancer states or chromatin maintenance in their own unique way, as these functions of *MED12* have not been thoroughly studied in relation to specific variants, rather only via knockout/knockdown models [2,39,40,42].

One more complicating factor regarding the different phenotypes associated with *MED12* is the role of X-inactivation. So far, studies looking at X-inactivation patterns in blood or fibroblast cells found no correlation between XCI skewing and phenotype severity. However, no studies have looked at XCI in tissues relevant to the pathology of neurodevelopmental disorders, such as brain tissues [1,2,39]. This is unfortunate, as we deem it likely that the difference in phenotypes observed for nonsense variants in females could be explained by differences in XCI patterns, where Hardikar patients have one healthy allele active in the brain, while patients suffering from neurodevelopmental disorders express the variant allele in neuronal tissue.

We propose that future studies on the effects of variants involved in *MED12*-related disorders should focus on answering these questions in a simple, disease-relevant cellular model. MED12 is known as an important factor in the regulation of enhancer and super enhancer functioning [1]. It regulates cell type-specific active enhancers through stimulation of active chromatin marks [2,40]. Disruption of MED12 leads to the collapse of enhancers [42]. We hypothesize that specific variants disrupt the ability of MED12 to regulate certain specific groups of genes. Unique disruption patterns of epigenetic markers might be associated with specific *MED12* variants. One study looking at epigenetic signatures in peripheral blood DNA of patients failed to find a specific signature for FG syndrome. However, this study was performed on a group of only nine patients, then only on peripheral blood DNA, and did not aim to identify differences between different *MED12*-related disorders [52].

**Figure 5:**
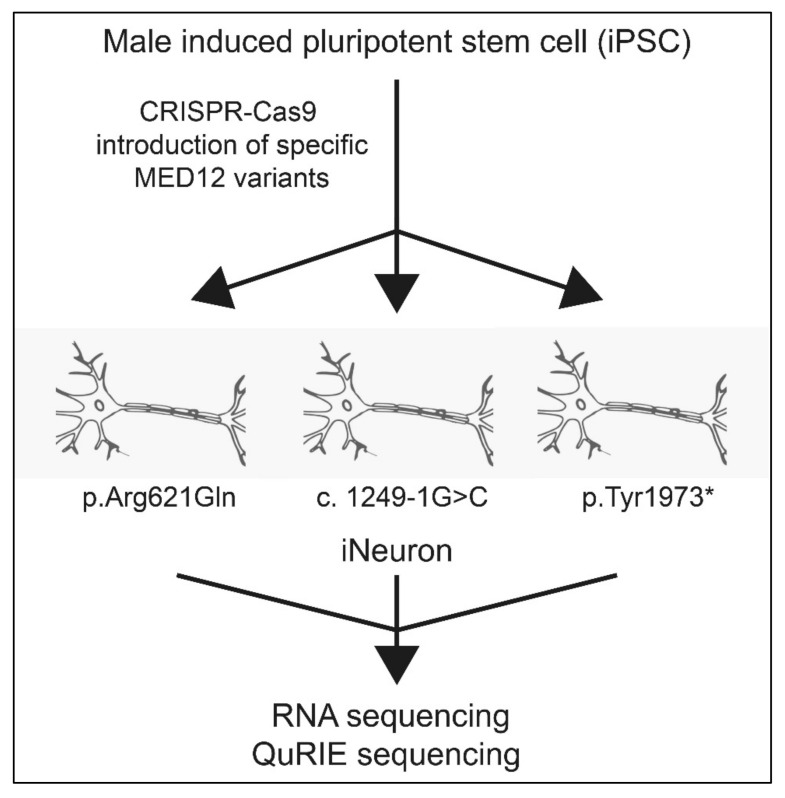
General approach to determine pathogenicity of MED12 variants. Firstly, an isogenic male iPSC is generated which also serves as a control. Then by use of CRISPR-Cas, the novel variants are introduced in the cells (e.g., p.Arg621Gln, c.1249-1G>C), pTyr1973*). These cell lines are subsequently sequenced and the differentially expressed genes as compared to a control are selected through an AI algorithm.

One way to analyze the effect of *MED12* variants in one step is to look at the transcription level of its effector genes. We would also need to get as close to neuronal tissue as is possible. The cell line used would ideally be healthy, induced pluripotent cells (iPSC), in which *MED12* variants could be introduced using gene editing, e.g., via CRISPR-Cas (Figure 5). The iPSC should be further transformed into an isogenic neuronal cell line (iNeuron) that mimics the neurons in the brain [53]. The technology for this is readily available. For the purpose of simplifying the model, a male cell line should be used, as this could also be used to as a model for a female cell in which X-inactivation is extremely skewed. The isogenic iNeuron cell model can then be used to answer important questions regarding the effects of different *MED12* variants. The different *MED12* variants all had a unique effect on the expression of immediate early gene expression [41] and it is expected that the other changes have a specific effect on gene expression as well. High-throughput omics techniques such as RNA sequencing or QuRIE sequencing (combining RNA seq with proteomics) can be used to gain an overview of general changes to the transcriptome or proteome after MED12 variants [54]. From these expression patterns, specific genes will be selected that are differentially expressed in the majority of the patients with *MED12* variants as compared to untransformed iNeuron cells. A relatively simple AI algorithm could help with this procedure. The expression profile of the differentially expressed genes then indicates whether a variant is pathogenic.

In conclusion, we propose an isogenic iNeuron model to discover the unique expression patterns that are associated with specific *MED12* variants. By looking first at the global expression of genes in iNeurons with a specific *MED12* variant, we expect to find a subset of genes that are differentially expressed in patients. The discovery of these patterns would help in future diagnostics and determine the causality of the *MED12* variants.

## Figures and Tables

**Table 1:** Comparison of phenotypes found in the kinase module genes. Intellectual disability was classified as mainly mild to moderate (+) or mainly moderate to severe (++). Other signs were considered as very frequent (+), occasional (+/−), or absent/rare (−). Lujan: Lujan-Fryns syndrome. FG: Opitz-Kaveggia (FG) syndrome. Ohdo: Ohdo Syndrome–Maat-Kievit-Brunner Type. This table was adapted from Nizon et al. 2019 [10], and supplemented with data from Calpena et al. 2019 [49] (CDK8), Chung et al. 2020 [5] (CDK19), and Li et al. [16] (Hardikar syndrome).

	*MED12*					*MED12L*	*MED13L*	*MED13*	*CDK8*	*CDK19*
	Non-Syndromic	Lujan	FG	Ohdo	Hardikar					
**Growth**										
Tall stature	-	+	-	-	-	-	-	-	-	-
Macrocephaly	+/−	+	+	-	-	-	-	-	+	-
Facies										
Tall prominent forehead	+	+	+	+	-	-	-	-	+/−	-
Blepharophimosis	+/−	-	-	+	+	-	-	-	-	-
Downslanting palpebrae	-	+	+	+	+	-	-	+/−	+/−	-
High nasal root	-	+	-	-	-	+/−	+	+	-	-
High narrow palate	+/−	+	+	+	+	+/−	-	-	+/−	+/−
Open mouth	-	+	+	+	-	-	+	-	+/−	-
Frontal hair upsweep	-	-	+	-	-	-	-	-	-	-
Hand										
Minor hand anomalies	-	+	+	+	-	+/−	+	-	+/−	-
Neorological										
Congenital hypotonia	+	+	+	+	+/−	+/−	+/−	+/−	+	+
Intellectual disability	++	+	+	++	-	+	++	+	++	+
Little or no language	++	-	-	+	-	-	+	+/−	-	+/−
Hypernasal voice		+	-	-	-	-	-	-	+/−	-
Behavior disturbances	+	+	+	+	-	+	+/−	+/−	+/−	+/−
Autism spectrum disorder	-	+/−	-	+/−	-	+/−	+/−	+/−	+/−	+/−
Agenesis/hypoplasia of corpus callosum	+	+	+	-	-	+	+/−	-	+/−	-
Gastrointestinal										
Anal anomalies	-	-	+	-	+/−	-	-	-	+/−	-
Chronic constipation	-	-	+	+	+/−	+/−	-	+/−	-	+/−

## Supplementary Materials

The following are available online at https://www.mdpi.com/article/10.3390/genes12050663/s1, Table S1: Previously published patients with a MED12 variant.
